# Centromeric association of small supernumerary marker chromosomes with their sister-chromosomes detected by three dimensional molecular cytogenetics

**DOI:** 10.1186/1755-8166-5-15

**Published:** 2012-03-14

**Authors:** Elisabeth Klein, Marina Manvelyan, Isabella Simonyan, Ahmed B Hamid, Roberta Santos Guilherme, Thomas Liehr, Tatyana Karamysheva

**Affiliations:** 1 Jena University Hospital, Friedrich Schiller University, Institute of Human Genetics, Kollegiengasse 10, D-07743 Jena, Germany; 2 Research Center of Maternal and Child Health Protection, Mashtots Ave. 22, 0002 Yerevan, Armenia; 3 Department of Genetic and Laboratory of Cytogenetics, State University, 1, Alex Manoukian Street, Yerevan, Armenia; 4 Laboratory of Morphology and Function of Cell structure, Institute of Cytology and Genetics, Russian Academy of Sciences, Siberian Branch, Lavrentiev Ave. 10, 630090 Novosibirsk, Russian Federation

## Abstract

**Background:**

Small supernumerary marker chromosomes (sSMC) are detected in 0.043% of general population and can be characterized for their chromosomal origin, genetic content and shape by molecular cytogenetic approaches. Even though recently progress was achieved towards genotype-phenotype-correlations of sSMC, nothing is known on the influence that an additional derivative extra chromosome has on the nuclear architecture.

**Results:**

Here we present the first three-dimensional interphase fluorescence in situ hybridization (FISH) studies for the nuclear architecture of sSMC. It could be shown that sSMC derived from chromosomes 15, 16 or 18 preferentially colocalized with one of their corresponding sister chromosomes. This was true in B- and T-lymphocytes as well as in skin fibroblasts. Additionally, a case with a complex sSMC with a karyotype 47,XY,+der(18)t(8;18)(8p23.2 ~ 23.1;18q11.1) was studied. Here the sSMC co-localized with one homologous chromosome 8 instead of 18.

**Conclusion:**

Overall, there is a kind of "attraction" between an sSMC and one of its homologous sister chromosomes. This seems to be transmitted by the euchromatic part of the sSMC rather than its heterochromatic one.

## Background

Small supernumerary marker chromosomes (sSMC) are reported in 0.043% of newborn infants, 0.077% of prenatal cases, 0.433% of mentally retarded patients and 0.171% of subfertile people [1]. They are defined as structurally abnormal chromosomes that cannot be identified or characterized unambiguously by conventional banding cytogenetics alone, and are generally equal in size or smaller than a chromosome 20 of the same metaphase spread. sSMC are mostly detected unexpectedly in routine cytogenetics [2] and are not easy to correlate with a specific clinical outcome [3]. It is known that ~30% of sSMC are derived from chromosome 15; ~11% are i(12p)-, i.e. Pallister-Killian-, ~10% are der(22)-, ~7% are inv dup (22)-cat-eye- and ~6% are i(18p)-syndrome associated sSMC [2]. Also essential progress towards genotype-phenotype-correlation was recently achieved [4,5].

Still little is known on the biology of sSMC in terms of formation [2,6], centromeric activity in dicentric sSMC [7], mitotic and meiotic stability [3] and coexistence of sSMC and uniparental disomy [8], or positioning of an sSMC in the interphase nucleus. Especially for the latter problem there were up to now only 'indirect' studies done, i.e. spread metaphases were analyzed to define the positions of the sSMC with respect to other chromosomes (summarized in [9]). In the most detailed study of those [9] an association of sSMC with centromeres of other chromosomes in between 27% and 41% was suggested.

The interphase nucleus recently became accessible for studies concerning the localization of chromosomes and chromosomal subregions; three-dimensional (3D) fluorescence in situ hybridization (FISH) analysis is the major tool for studying the higher order chromatin organization in the cell nucleus [10-16]. In this connection, chromosome size and gene density are discussed to have an impact on the nuclear position of chromosomes [15]. Furthermore, non-random positioning in interphase nuclei is nowadays known to be of importance for genomic stability as well as for formation of chromosomal aberrations [16]. Here we apply for the first time 3D-interphase FISH to characterize the position of sSMC with respect to their sister chromosomes.

## Results

3D-interphase FISH [12] using whole (wcp) and partial chromosome painting (pcp) [17] as well as commercially available centromeric probes (cep) was applied in five sSMC (cases 1 to 5) and one normal control case (case 6 - see Table 1). B-lymphocytes (cases 3 to 5), T-lymphocytes (cases 1 and 6) and skin fibroblasts (case 2) were studied. The sSMC were derived from chromosomes 15 (cases 1 and 2), 16 (case 3) and 18 (cases 4 and 5). In case 5 a so-called complex sSMC [18], i.e. consisting of parts derived from chromosomes 8 and 18, was studied.

**Table 1:** Used sSMC cases and controls, their karyotypes, the studied material and ID of the cell line

case number	karyotype	studied material	cell line ID
1	47,XX,+inv dup(15)(q11.1)	T-lymphocytes	n.a. but case listed as 15-O-q11.1/1-70 in [5]

2	47,XX,+inv dup(15)(q12)	skin fibroblasts	Coriell GM07992

3	47,XY,+min(16)(:p11.1- > q12.1)	B-lymphocytes	EKF-#16-p11.1/1-m

4	47,XY,+inv dup(18)(q11.1)	B-lymphocytes	EKF-#18-q11.1/1-i

5	47,XY,+der(18)t(8;18)(8p23.2~23.1;18q11.1)	B-lymphocytes	EKF-#18compl#8/1-m

6	46,XX	T-lymphocytes	n.a. - control

Abbreviations: Coriell = Coriell institute http://ccr.coriell.org/Sections/Search/Sample_Detail.aspx?Ref=GM07992&PgId=166; EKF = Else Kröner-Fresenius-cellbankfor small supernumerary marker chromosomes http://www.fish.uniklinikum-jena.de/sSMC/sSMC+by+chromosome/EKF_cellbank.html

**Figure 1:**
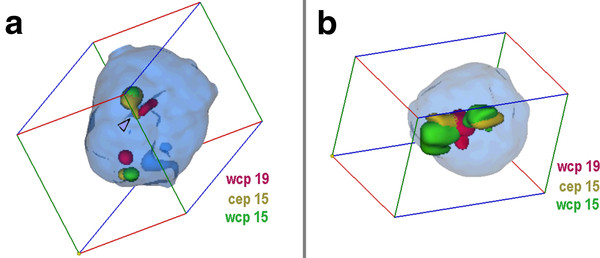
**Nuclei with and without sSMC(15) derived from T-lymphocytes**. A) Representative interphase nucleus for case 1. In the figure the position of the sSMC is highlighted (arrowhead) and the pseudocolors together with the applied probes are given. One chromosomes 19 is central, the other peripheral positioned, both chromosomes 15 have similar positions as chromosomes 19. The sSMC is colocalized with the chromosomes 15 and 19 in central position. B) Representative interphase nucleus of case 6 using probe set for case 1. Chromosomes 19 are in close together and central, chromosomes 15 are in central to intermediate positions. N.B. Animations of the corresponding nuclei are available as additional file - film 1.

**Figure 2:**
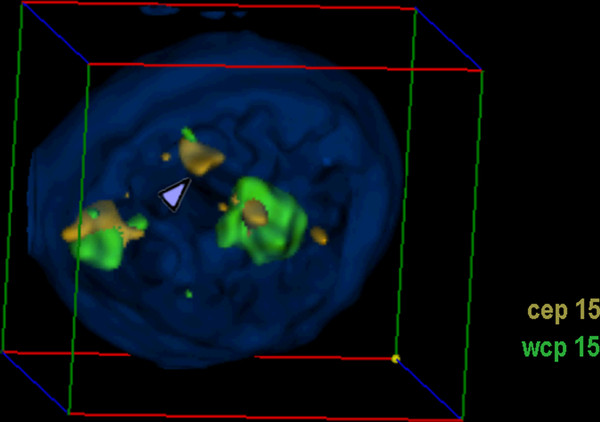
**Nucleus with sSMC(15) derived from skin fibrobloast**. Representative interphase nucleus for case 2, shown in another depiction than in other figures, and not showing chromosome 19 probe. In the figure the position of the sSMC is highlighted (arrowhead) and the pseudocolors together with the applied probes are given. N.B. An animation of the corresponding nucleus is available as additional file - film 2.

**Figure 3:**
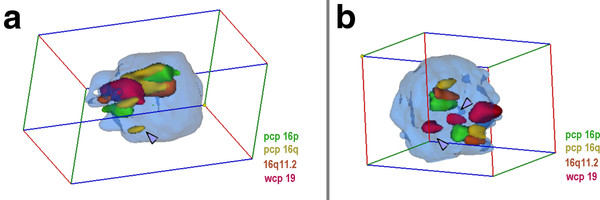
**Nuclei with and without sSMC(16) derived from lymphocytes**. A) Representative interphase nucleus for case 3. In the figure the position of the sSMC is highlighted (arrowhead) and the pseudocolors together with the applied probes are given. Chromosomes 19 are in close together and intermediate to peripheral, and both chromosomes 16 and the sSMC are intermediate to peripheral positioned. The sSMC is not colocalized with any of the tested chromosomes. **B) **Representative interphase nucleus of case 6 using probe set for case 3. One chromosome 19 is in central position and appears as two separate spots due to image processing (arrowheads) and the other chromosome 19 is in intermediate to peripheral position. One chromosome 16 is in central to intermediate, the other in peripheral to intermediate position. N.B. Animations of the corresponding nuclei are available as additional file - film 3.

**Figure 4:**
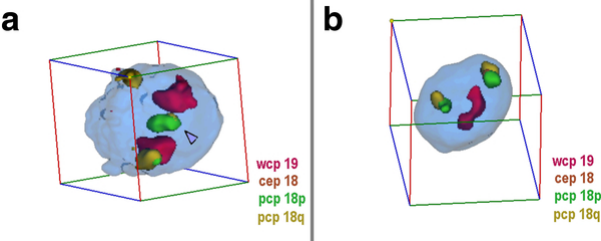
**Nuclei with and without sSMC(18) derived from lymphocytes**. A) Representative interphase nucleus for case 4. In the figure the position of the sSMC is highlighted (arrowhead) and the pseudocolors together with the applied probes are given. Chromosomes 19 are in intermediate to peripheral positions, chromosomes 18 in peripheral and opposite. The sSMC is in central position. B) Representative interphase nucleus of case 6 using probe set for case 4. Chromosomes 19 are in central position and close together. Both chromosomes 18 are in peripheral and opposite. N.B. Animations of the corresponding nuclei are available as additional file - film 4.

**Figure 5:**
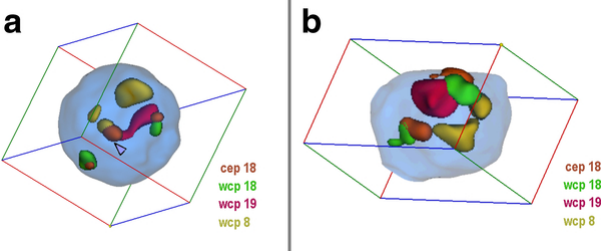
**Nuclei with and without complex sSMC(18) derived from lymphocytes**. A) Representative interphase nucleus for case 5. In the figure the position of the sSMC is highlighted (arrowhead) and the pseudocolors together with the applied probes are given. Chromosomes 19 are in close together and central, chromosomes 8 and 18 are in opposite orientation and peripheral positions, the sSMC is in central to intermediate position and colocalized to a chromosome 19. B) Representative interphase nucleus of case 6 using probe set for case 5. Chromosomes 19 are in close together and central to intermediate, chromosomes 8 and 18 are in peripheral and opposite positions. N.B. Animations of the corresponding nuclei are available as additional file - film 5.

**Figure 6:**
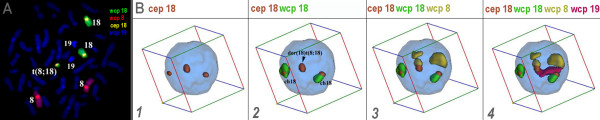
**The used probe sets were established and tested on normal control and in metaphase of the studied case itself (A)**. Afterwards the probe sets were used in S-FISH analysis **(B)**.

The applied probe sets were tested on normal metaphase spreads first and are shown in Figures 1, 2, 3, 4, 5 and 6 and Additional files 1, 2, 3, 4 and 5; each probe set was tested on control case 6. A wcp 19 probe served as internal control in all experiments.

The position of the sSMC itself within in the nucleus was more central to intermediate in cases 1, 2, and 4, and peripheral to intermediate (cases 3 and 5). sSMC derived from chromosomes 15 and 18 were located more central, those derived from chromosomes 16 and 8/18 more peripheral (Table 2).

**Table 2:** Position of sSMC within the nucleus.

position Case	P - periphery [%]	I - intermediate [%]	C - central [%]	number of nuclei
1 (#15)	17	48	35	40

2 (#15)	4	26	70	23

t-test	*P *< 0.002	*P *< 0.003	*P *= 0.001	n.a.

3 (#16)	46	27	27	15

4 (#18)	21	28	51	68

5 (#18)	47	45	8	36

t-test	*P *< 0.002	*P *= 0.001	*P *< 0.001	n.a.

Comparison of cases 1 and 2 and 4 and 5, respectively, by *t*-test. Abbreviations: n.a. = not available, nsd = no significant difference

As expected, both chromosomes 19 were located predominantly in a central position and this was not altered due to sSMC presence in any case or tissue. Positioning of chromosomes 8, 15 and 18 with respect to central or peripheral orientation was also not influenced by sSMC presence (Table 3): chromosomes 8 and 18 were located more in the nuclear periphery; chromosome 15 was more in an intermediate position. Chromosome 16 was normally also in an intermediate position; interestingly, the position of normal chromosomes 16 was shifted significantly towards the periphery in sSMC(16) cells (Table 3).

**Table 3:** Position of normal chromosomes 8, 15, 16, 18 and 19 in cells with and without an sSMC.

	P - periphery [%]			I - intermediate [%]			C - central [%]			number of nuclei	
**case**	**no sSMC**	**with sSMC**	**t-test**	**no sSMC**	**with sSMC**	**t-test**	**no sSMC**	**with sSMC**	**t-test**	**no sSMC**	**with sSMC**

1	21	32	nsd	45	42	nsd	34	26	nsd	42	84

2	n.a.	44	n.a.	n.a.	31	n.a.	n.a.	40	n.a.	n.a.	36

3	28	53	*P *< 0.001	42	24	*P *< 0.002	30	23	*P *= 0.007	40	52

5	63	55	nsd	25	39	yes *P *= 0.002	12	6	nsd	52	69

4 and 5	65	61	nsd	27	31	nsd	8	8	nsd	52	108

1 to 5	12	13	nsd	38	39	nsd	50	48	nsd	133	274

Abbreviations: n.a. = not available, nsd = no significant difference

The distance and orientation of the homologous chromosomes to each other was influenced by sSMC presence (Table 4). In all cases there was a tendency that the homologous sister-chromosomes where more separated from each if an sSMC was present: in all five cases there were significantly more nuclei observed with opposite orientation of the sister chromosomes than when no sSMC was present. In case 2 the normal (close) together orientation of homologous chromosomes was almost completely replaced by nearby or opposite orientation. Strikingly, the orientation of both chromosomes 19 to each other was not influenced at all by sSMC presence (Table 4); if this would change in case of an sSMC(19) remains to be tested.

**Table 4:** Orientation of normal chromosomes 8, 15, 16, 18 and 19 to each other in cells with and without an sSMC.

	T - together [%]			N - nearby [%]			O - opposite [%]			number of nuclei	
**case**	**no sSMC**	**with sSMC**	**t-test**	**no sSMC**	**with sSMC**	**t-test**	**no sSMC**	**with sSMC**	**t-test**	**no sSMC**	**with sSMC**

1	43	56	*P *< 0.003	57	36	nsd	0	8	*P *< 0.001	42	78

2	n.a.	4	n.a.	n.a.	82	n.a.	n.a.	4	n.a.	n.a.	23

3	42	40	nsd	58	51	nsd	0	9	*P *< 0.001	26	79

5	28	30	nsd	66	17	*P *< 0.001	6	53	*P *< 0.001	100	41

4 and 5	36	28	*P *< 0.006	52	36	*P *< 0.002	12	36	*P *< 0.002	50	71

1 to 5	28	12	*P *< 0.002	66	62	nsd	6	26	*P *= 0.001	100	69

	67	73	nsd	31	25	nsd	2	2	nsd	174	259

Abbreviations: n.a. = not available, nsd = no significant difference

Finally, as visible in Table 5, the sSMC were orientated towards/colocalized with one of their corresponding sister-chromosomes. Chromosome 19 served here as well as a control - a random colocalization of the sSMC and one of chromosomes 19 was observed in 6-30% of the cells. For sSMC(15) this kind of nearby orientation was more expressed in fibroblasts (87%) than in T-lymphocytes (58%). The colocalization of sSMC(16) and sSMC(18) with chromosomes 16 and 18, respectively, in cases 3 and 4 was also non-random with 52 and 53%. In case 5, with the complex sSMC composed from chromosomes 8 and 18, there was a random colocalization of the sSMC with one chromosome 18 (27%) but a non-random one with chromosome 8 (56%). As chromosome 8 has a more peripheral position and chromosome 19 a central one, the colocalization with the latter chromosome is only 6% here.

**Table 5:** Colocalization of sSMC with sisterchromosome (SC).

position case	colocalized SC [%]	separate SC [%]	colocalized #8 [%]	colocalized #19 [%]	number of nuclei
1 (#15)	58	18	not tested	24	43

2 (#15)	87	13	not tested	not tested	23

t-test	*P *< 0.001	nsd	n.a.	n.a.	n.a.

4 (#18)	53	17	not tested	30	36

5 (#18)	27	11	56	6	35

t-test	*P *< 0.001	no	n.a.	*P *< 0.001	n.a.

Comparison of cases 1 and 2 and 4 and 5, respectively, by *t*-test. Abbreviations: n.a. = not available, nsd = no significant difference

## Conclusion

Using 3D-interphase FISH applying wcp, pcp and cep probes in five sSMC-cases led to new interesting insights into how the nuclear architecture is influenced by presence of an additional extra chromosome. The most important result is that this extra piece of DNA leads to gross rearrangements within the interphase nucleus. As the nuclear architecture is considered to be important for correct DNA-transcription, gene-regulation and -expression [19] this is an important finding.

Taking together all results summarized in Tables 1, 2, 3, 4, 5 there is evidence that the position of the sSMC is primarily influenced by and/or influencing the position of the homologous chromosomes. In cases 1 and 2 the sSMC derived from chromosome 15 were, as their sister-chromosomes, in a more central position, and in case 5 the sSMC derived from chromosomes 8 and 18 was predominantly in the periphery, like chromosomes 8 (Table 2). Even though chromosomes 18 are located in nuclear periphery the sSMC(18) in case 4 was most often in an intermediate to central position (Table 2). The same in reverse holds true for chromosomes 16 (central to intermediate position) and the sSMC(16) in case 3 (more peripheral localization). This could be due to the relative amount of genes present on the sSMC: the min(16)(:p11.1- > q12.1) in case 3 has almost no gene content and thus, could be driven towards periphery, while the inv dup(18)(q11.1) from case 4 has compared to its size more genes and could thus be driven to the central part of the nucleus [15].

Nonetheless, the sSMC are orientated in the majority of the studied cells to one of their corresponding sister-chromosomes (Table 5). In case 2 the colocalization of the sSMC(15) and one sister-chromosome was observable in even 87% of the cells. This is much higher than in case 1, with an sSMC(15) as well. The difference can be due to two reasons: either there is another nuclear architecture in fibroblasts (case 2) than in T-lymphocytes (case 1), or there is an influence of the size of the euchromatic content on the sSMC and the colocalization rate; the sSMC in case 2 has much more euchromatin derived from 15q than that of case 1, which is pure heterochromatin. It needs further studies to elucidate this point; however, it is noteworthy that in case 5 the euchromatic part of the complex sSMC seem to drive the colocalization to a chromosome 8 rather than chromosome18.

Deducing from the data for position of chromosome 19 in this study, mainly sister-chromosomes are influenced in their 3-D-positioning by sSMC-presence (Tables 3 and 4). In general the positioning of the sSMC's sister-chromosomes is not affected by the sSMC presence (Table 3). Only in case 3 there is an exception and the position of normal chromosomes 16 is altered from an intermediate to a more peripheral position. Here the more peripheral position of the sSMC(16) seems to drive the homologous chromosomes 16 also towards periphery.

According to the data summarized in Tables 4 and 5 the preferred colocalization of an sSMC and its sister-chromosome could be interpreted in that way, that the sSMC tends to take over the position of the normal sister-chromosome. Thus, the remainder sister-chromosome is displaced towards another location within the nucleus. This is reflected by the fact that in all studied sSMC-cases there was a tendency that the homologous sister-chromosomes where more separated from each if an sSMC was present (Table 4).

Overall, this study highlights the necessity to perform more 3D-FISH studies in cases with chromosomal gains and rearrangements. New insights in reshufflings of genomic parts could alter our understanding of the effects of cytogenetic changes for the cellular expression.

## Material and methods

### Interphase cells

In the present study interphase cells were used, prepared according to standard procedures for chromosome harvesting [16]. Interphases were obtained from lymphoblastoid cell lines, peripheral blood cells or skin fibroblasts (Table 1). All karyotypes were determined previously by cytogenetic standard banding and different molecular cytogenetic approaches as summarized for each case in [5].

### Molecular cytogenetics

#### Suspension FISH (S-FISH)

Wcp, pcp and cep for chromosomes 8, 15, 16, 18 and/or 19 [17] were applied together in suspension-FISH (S-FISH) as previously reported [12-16]. Images of 3D-preserved interphase nuclei were captured on a Zeiss Axioplan microscope and analyzed by the Cell-P (Olympus) software. 50-70 interphase nuclei were acquired and processed per case. Between 30 and 40 nuclei were evaluated as reported previously [14-16].

#### Evaluation

For the 3D-evaluation, position and distance of homologous chromosomes were determined. The interphase nucleus was divided into two spheres, i.e. periphery (P) and center (C); 50% of the nucleus radius was defined as 'center'. Thus, analyzed chromosomes could be allocated either as C or P. Similar as described in [14] the relative positions of the studied chromosomes to each other were recorded as 'close together' (t), 'near by each other' (n) or 'on the opposite sides of the nucleus' (o) for two homologue chromosomes - for examples see Figures 1, 2, 3, 4, 5 and 6 and Additional files 1, 2, 3, 4 and 5.

#### Statistics

Statistical analysis was performed using Student's t - test, One Way ANOVA (Analysis of Variance) and Holm-Sidak method. Statistical significance was defined as *p *< 0.05.

## Competing interests

The authors declare that they have no competing interests.

## Authors' contributions

TK, EK, ABH, RdSG and MM participated in the molecular cytogenetics studies. TL drafted and edited the manuscript. All authors read and approved the final manuscript.

## Supplementary Material

Additional file 1 **Film 1. Nuclei with and without sSMC(15) derived from T-lymphocytes**. In the first part of the film of a representative interphase nucleus for case 1 is visible - to better localize the sSMC see Figure 1. In the second part a representative interphase nucleus of case 6 using probe set for case 1 is presented. Click here for file

Additional file 2 **Film 2. Nucleus with sSMC(15) derived from skin fibrobloast**. Representative interphase nucleus for case 2, - to localize the sSMC see also Figure 2. Click here for file

Additional file 3 **Film 3. Nuclei with and without sSMC(16) derived from lymphocytes**. In the first part of the film of a representative interphase nucleus for case 3 is visible - to better localize the sSMC see Figure 3. In the second part a representative interphase nucleus of case 6 using probe set for case 3 is presented. Click here for file

Additional file 4 **Film 4. Nuclei with and without sSMC(18) derived from lymphocytes**. In the first part of the film of a representative interphase nucleus for case 4 is visible - to better localize the sSMC see Figure 4. In the second part a representative interphase nucleus of case 6 using probe set for case 4 is presented. Click here for file

Additional file 5 **Film 5. Nuclei with and without complex sSMC(18) derived from lymphocytes**. In the first part of the film of a representative interphase nucleus for case 5 is visible - to better localize the sSMC see Figure 5. In the second part a representative interphase nucleus of case 6 using probe set for case 5 is presented. Click here for file
